# Profiles of Adolescent Identity at the Intersection of Ethnic/Racial Identity, American Identity, and Subjective Social Status

**DOI:** 10.3389/fpsyg.2020.00959

**Published:** 2020-05-15

**Authors:** Yuen Mi Cheon, Pak See Ip, Milou Haskin, Tiffany Yip

**Affiliations:** ^1^Department of Child Development and Education, Myongji University, Seoul, South Korea; ^2^Department of Psychology, Fordham University, New York, NY, United States

**Keywords:** intersectionality, social identity, ethnic/racial identity, American identity, subjective social status, ethnic/racial minority adolescents

## Abstract

Ethnic/racial minority adolescents face the task of forming an identity in relation to their ethnic/racial group as well as to American society, while also developing awareness of their social status relative to salient social groups. Whereas previous studies have investigated individual social identity dimensions or examined how objective measures of ethnicity/race and socioeconomic status intersect, studies that take a holistic and person-centered approach to considering various configurations of multiple social identities with subjective measures have been less common. The current study addresses these gaps and explores profiles of ethnic/racial identity, American identity, and subjective social status among ethnic/racial minority adolescents. Next, differences in discrimination experiences, mental health and academic outcomes across these profiles were examined. Three distinct identity profiles emerged from the data – “weakly identified,” “high ethnic/racial identity moderate American identity,” and “moderate ethnic/racial identity and American identity.” The “weakly identified” demonstrated the highest levels of past discrimination experiences and depressive symptoms, while the “moderate ethnic/racial identity and American identity” group reported the lowest levels of school engagement. Interpretation of the profiles and associated outcomes and implications are discussed.

## Introduction

During adolescence, individuals become aware of their membership in various social groups through dynamic interactions with the expanding developmental environment (Erikson, 1960; Bronfenbrenner, 1992). As individuals adopt these group memberships into the formation of their own identity, they begin to form social identities (Tajfel and Turner, 1979). In ethnically/racially diverse contexts, the development of identity takes place against the backdrop of the broader society and the value that society places on one’s social group membership. This is concerning because social identity theory (Tajfel and Turner, 1979) posits that a strong and positive identification with one’s social group is an important source of positive self-image. In addition, the formation of identity is associated with positive mental health and academic outcomes (Tajfel and Turner, 1986; Cameron, 1999; Smith and Silva, 2011; Rivas-Drake et al., 2014; Reynolds et al., 2017).

Each individual possesses a unique configuration of social identity dimensions developed across multiple contexts (Luyckx et al., 2014). In the United States, adolescents’ ethnic/racial communities and the American society are both important contexts for developing social identity dimensions such as an ethnic/racial identity (ERI; Phinney et al., 1997; Sue and Sue, 2013) and an American identity (Devos and Sadler, 2019; Tikhonov et al., 2019). There is a developing understanding of the relative social status of each ethnic/racial group that parallels the development of ERI and social identity more broadly (Goodman et al., 2001, 2015; Sani and Bennett, 2004; Kiang et al., 2008). Moreover, ERI, American identity, and social status have each been found to be related to disparities in health and academics (Brondolo et al., 2009; Williams and Sternthal, 2010; Sims and Coley, 2019). While these studies have depicted ERI, American identity, and social status as distinct social identity dimensions, each playing a unique role in adolescent development, different configurations of these dimensions – when they are considered together – differentially inform various developmental outcomes (Chatman et al., 2001; Ashmore et al., 2004; Rowley et al., 2007; Goodman et al., 2015; Evans and Erickson, 2019). In fact, unique configurations, or the *intersection*, of these multiple identity dimensions is likely to capture adolescent development more accurately than focusing solely on a single dimension of social identity (Hancock, 2007; Cole, 2009; Syed and Azmitia, 2010; Azmitia and Thomas, 2015; Rogers, 2019).

The term intersectionality was initially developed to consider the intersections of various demographic factors (e.g., race, class, and gender) in the experiences of oppression (Crenshaw, 1991). An intersectional perspective allows for the unique discovery of configurations of multiple marginalization and/or privilege statuses (Bauer, 2014; Evans et al., 2018; Wolff et al., 2010). Previously, the concept of identity intersectionality has often been operationalized with objective measures of ethnicity/race and socioeconomic status (Wolff et al., 2010; Gong et al., 2012; Goodman et al., 2015), and less research has focused on subjective perceptions of identity intersectionality, despite another line of research suggesting the importance of subjective perceptions of one’s place in a society (Deaux, 1996; Zell et al., 2018).

The present study takes an intersectional approach and pays attention to the subjective perceptions of social identity dimensions. Specifically, to gain a holistic understanding of the adolescents in the current study, the profiles of ethnic/racial minority adolescents’ social identity configurations across ERI, American identity, and subjective social status (SSS) are explored. Next, differences in the experience of disadvantage (i.e., discrimination) and subsequent developmental outcomes (i.e., mental health and academic achievement) are examined across the identified identity profiles.

One of the social identity dimensions included in the exploration of profiles is adolescents’ ERI. ERI consists of a wide range of constructs that describe how individuals feel and think about their ethnic/racial group memberships (Umaña-Taylor et al., 2014). Previous developmental theories of identity (Marcia, 1966; Erikson, 1968; Phinney, 1989; Sellers et al., 1998) have discussed two dimensions of ERI: content and process. While the content of one’s ERI includes attitudes and beliefs about one’s ethnic/racial group membership, the process of developing one’s ERI includes exploration and commitment to one’s ethnic/racial group. The current study focuses on the exploration and commitment processes of ERI development. Exploration refers to efforts of thinking and searching for the meanings of one’s ethnic/racial group. Individuals may talk to others from their ethnic/racial group or participate in cultural events to seek information and knowledge about their ethnic/racial group. Commitment refers to the life choices and level of attachment an individual has to their ethnic/racial group. The existing studies suggest that different levels of ERI exploration and commitment result in both positive and negative developmental outcomes. Adolescents who reported different levels of ERI affirmation and resolution have reported different levels of discrimination experiences (Zeiders et al., 2019). Adolescents whose ERI was under-developed displayed higher levels of depression and anxiety, lower levels of school engagement and academic grades (Donovan et al., 2013; Sanchez et al., 2016; Tabbah et al., 2016). In other instances, ERI commitment serves as a protective factor against psychological distress, while ERI exploration functions as a risk factor (Torres and Ong, 2010; Torres et al., 2011; Yip et al., 2019b). Although higher levels of ERI are generally viewed as beneficial for development, this is not always the case. Further, the relationship between developmental outcomes and ERI, when considered alongside other social identity dimensions, such as American identity and SSS, is largely unexplored.

In addition to ERI, American national identity is an important dimension of social identity for youth in the United States. In the United States, American identity is seen as a national identity and often associated with “whiteness” or a White social status (Devos and Sadler, 2019; Tikhonov et al., 2019). Unfortunately, ethnic/racial minority individuals continue to be considered perpetual foreigners, experiencing discrimination and identity denial. The importance of an American identity for ethnic/racial minority individuals is underscored in research which finds that being denied one’s American identity leads to greater negative emotions, higher rates of identity threat, greater depression, lower hope, and life satisfaction (Huynh et al., 2011; Wang et al., 2012; Sanchez et al., 2018). In fact, denial of one’s American identity is associated with an increased effort to assert one’s belonging to American society (Cheryan and Monin, 2005). All of these studies implicate that having a strong American identity may be helpful for adolescents’ development. However, again, these studies have not examined American identity with other social identity dimensions – how various social identity dimensions may operate together as different configurations. For example, high levels of American identity accompanied by low levels of ERI may have different developmental implications from high levels of American identity accompanied by high levels of ERI.

An intersectional exploration of both ERI and American identity will provide developmental implications for different combinations of these two dimensions of identity. Research on racial/cultural identity formation details processes whereby ethnic/racial minority individuals maneuver through stages of ERI development where they first deny their ethnic/racial minority identity and follow the mainstream “American” culture (conformity stage). Next, they experience conflict in their feelings of Americanness and belonging to the ethnic/racial group (dissonance stage). Then, they deny the mainstream “American” culture to explore their minority identity (resistance and immersion stage), and through observing their rigidity in adhering to the ethnic/racial culture, individuals start to explore other cultures, still experiencing conflicts and learning to selectively trust the dominant society (introspection stage). Eventually, they may form a balanced identity – a configuration with high levels of both ethnic/racial minority group membership and the mainstream “American” group membership (integrative awareness stage; Sue and Sue, 2013). Although the achievement of a balanced identity is deemed ideal, movement across these stages vary by individuals and many may never reach the final stage. These stages are hypothesized to be related to health and developmental outcomes. For example, a configuration depicted by weak identification with both ethnic/racial group and American national group (dissonance stage) may have negative impact on development. On the other hand, moderate identification with both groups (introspection), strong identification with one or the other (conformity or resistance and immersion stage), or with both (integrative awareness stage), may have qualitatively different implications for adolescents’ development. Since Sue and Sue (2013) suggest that the achievement of a balanced identity is the ideal state, adolescents identify with having a combination of high levels of ERI and high levels of American identity can be hypothesized to display the most favorable developmental outcomes, while those who demonstrate a combination of weak ERI and American identity are expected to display the least favorable developmental outcomes.

Both ERI and American identity develop alongside perceptions of SSS – another social identity dimension. Adolescence is a developmental period when individuals become sensitive to their social standing relative to those around them. SSS is “a person’s belief about [their] location in a status order” (Davis, 1956), and is distinguished from objective measures of social status, such as income and education levels, in that it captures a wider scope of an individual’s self-perception. Jackman (1979) found that subjective status is commonly interpreted as both a social and economic construct. It includes the individual’s current social standing, their background, and their perception of the opportunities in their future, which are shaped by the socioeconomic, educational, and ethnic/racial elements of their background (Singh-Manoux et al., 2003). SSS is a well-established predictor of both mental and physical health (Zell et al., 2018), wherein individuals reporting higher SSS are found to be healthier than those who report lower SSS (Quon and McGrath, 2014). Not only does the association between SSS and health remain after controlling for traditional measures of socioeconomic status (Cundiff and Matthews, 2017), SSS has been found to be more strongly related to self-rated health, chronic stress, sleep latency, and other physical health and psychological outcomes, such as obesity, depression, and subjective well-being, than objective status (Adler et al., 2000; Singh-Manoux et al., 2005).

Social stratification and comparison, such as stigma, discrimination, and other forms of societal perceptions of social groups that may be factored into one’s relative social placement, not only play a critical role in understanding well-being and social identity, but also complement objective measurements. In the United States, issues of ERI, American identity, and social status are inextricably intertwined (Crenshaw, 1991; DeNavas-Walt and Proctor, 2014) as evidenced by many studies that support ethnic/racial disparities in social status (Losin et al., 2014; Goodman et al., 2015). Past studies have documented close relationships between various dimensions of social identity (e.g., ERI, American identity, and social status) and adolescents’ development, such as mental health and academic experiences (Chavous et al., 2003; Nitardy et al., 2015; Vaghela and Ueno, 2017; Piña-Watson et al., 2018; Constante et al., 2019; Rivenbark et al., 2019). The findings of these studies suggest that while ERI, American identity and SSS are distinct social identity dimensions, it would also be important to take an intersectional approach and consider different combinations of these social identities.

One value of taking an intersectional approach of considering various combinations of social identity dimensions is that it uniquely elucidates marginalization that is experienced in multiple spaces; with the working hypothesis that multiple areas of marginalization are likely related to amplified vulnerabilities. In an attempt to unpack social inequality, scholars have adopted an intersectionality framework to consider the independent and joint effects of multiple social positions such as socioeconomic status and marginalization statuses; as well as between-group homogeneity and within-group heterogeneity (Azmitia and Thomas, 2015). Past studies have highlighted socioeconomic status and ethnicity/race as two important axes of social identity among adults (Wolff et al., 2010; Goodwin et al., 2018). Recent studies of intersectionality have found that different configurations of intersecting and competing identities contribute to disparities in developmental, social, and academic outcomes, reiterating the usefulness of a person-centered approach (Shade et al., 2011; Williams et al., 2012). However, the current body of literature is short on quantitative operationalizations of “intersectionality of identity,” especially among adolescents (Block and Corona, 2014) – a gap the current study seeks to address. By taking a quantitative approach, different subgroups can be identified by various configurations of multiple indicators that can be compared with one another. It becomes possible to quantify the average characteristics of these qualitatively different subgroups. Furthermore, categorizing the participants into these different subgroups is expected to provide information about the proportion of each subgroup that may be potential targets of prevention and intervention (Zeiders et al., 2013).

Intersectionality also has the potential to reveal important within-group differences. Within the same socioeconomic status and same ethnic/racial group, an individual’s sense of ethnic/racial and American identities may differ. For example, while a Latinx adolescent may perceive oneself as having a low social status in the United States, they may feel strongly attached to an ethnic/racial group and American identity. Another Latinx adolescent may also perceive oneself as having a low social status in the United States, have a weak attachment to an ethnic/racial group, but a strong attachment to an American identity. The current study takes a person-centered approach, and seeks to identify various configurations of social identity dimensions that exist among the ethnic/racial minority adolescents with ERI, American identity, and social status. We next examine whether these identity configurations are reflected in differences in prior experiences of discrimination, as well as subsequent mental health and academic outcomes. The three overarching research questions of the current study are:

RQ1: What profiles of adolescent identity will emerge at the intersection of ERI, American identity, and SSS? Are there differences in the presence of these profiles across ethnic/racial groups?
RQ2: How do profiles of adolescent identity differ in reports of previous discrimination?
RQ3: How are profiles of adolescent identity prospectively related to developmental outcomes 6 months later?

## Materials and Methods

### Participants

The present study utilizes first-year data from a larger 3-year study in which ninth grade student’s from five ethnically/racially diverse New York City public high schools were recruited for a study on identity development. The initial sampling took place at the school level. Schools were chosen based on the diverse student population, as determined by the New York City Department of Education, and letters were sent to principals to invite their institutions to participate. The participating schools were located in three different boroughs: Bronx, Brooklyn, and Queens. Researchers recruited ninth grade students at the schools via in-class presentations and flyers. Consent forms were mailed to the parents of eligible adolescents. Of the 405 participants in the original study, participants were included in the present sample based on self-reported primary race. Adolescents who reported their primary race as Asian, Black, or Latinx were included, while participants who reported White as their primary race were excluded; yielding data from 350 ethnic/racial minority adolescents of Asian (41%), African American (22%), and Latinx (37%) backgrounds. Of these adolescents, 24.3% (*n* = 85) considered themselves to be a member of more than one racial/ethnic group and 75.1% (*n* = 263) did not. The sample includes 242 females (69.1%) and 108 males (30.9%) between the ages of 13–17, with a mean age of 14.27 years (*SD* = 0.61). Most of the adolescents were born in the United States (*N* = 178, 51.45%), while another large percentage of the participants chose not to disclose their nativity (*N* = 113, 32.66%).

### Procedure

Participants completed an online survey three times during each school year: early fall, mid-fall, and spring. Students were given a tablet, or sent an email or text message with the survey link at each time point. A total of nine time points were included in the 3-year study in order to follow developmental patterns over the course of the years. The surveys distributed at these time points included similar instruments to assess well-being and academic outcomes, however, some of the measures differed by time point. Because the second and third years were not yet available for analysis at this time, only the first-year data was included. Considering the differences in measures at different time points and in order to provide implications for potential future longitudinal studies with the available dataset, all three time points within the first year were included. For the examination of how prior experiences of discrimination may have implications for different identity configurations, discrimination reports from the fall of the preliminary year of the study were used. Their ERI, American identity, and SSS were taken from the survey that was conducted 2 weeks later. In order to provide implications for subsequent mental health and academic outcomes, participants’ reports on depressive symptoms, anxiety, self-esteem, school adjustment, and average grades were collected from the data measured 6 months later. All procedures were approved by the Internal Review Board of the institution where the study was conducted.

### Measures

*Ethnic/Racial Identity Exploration and Commitment*. Participants’ ERI exploration and commitment were measured by the Multidimensional Ethnic Identity Measure (Phinney, 1992). Exploration included seven items such as “I have spent time trying to find out more about my ethnic group, such as its history, traditions, and customs.” Commitment included seven items such as “I feel a strong attachment toward my own ethnic group.” The responses were coded as “strongly agree” = 0, “agree” = 1, “disagree” = 2, “strongly disagree” = 3 (*M*_exploration_ = 1.67, *SD*_exploration_ = 0.57; *M*_commitment_ = 2.03, *SD*_commitment_ = 0.54). The Cronbach’s alpha of Exploration was 0.74 and Commitment was 0.86.

*American Identity*. Participants’ sense of American national identity was measured by the American identity measure (AIM) developed by Schwartz et al. (2012). A total of seven items, such as “How much did you feel like an American?” were asked on a 4-point Likert scale (“strongly disagree” = 1, “strongly agree” = 4). The reliability of this scale was 0.90.

*Subjective social status*. SSS was measured by the McArthur Scale of Subjective Socioeconomic Status (Adler et al., 2000). Participants were asked to mark their position relative to others in five different groups (i.e., the United States, school, age group, ethnic/racial group, and community). Originally the youth version of the McArthur Scale only included the contexts of the United States and school, but the current study modified this scale by including the community, which was included in the adult version of the scale, as well as their age group and ethnic/racial group in order to address multiple social contexts that may be relevant to ethnic/racial minority adolescents’ lives. The participants were asked to mark their position for each social group, five times in total, in the same order. Responses ranged from “top of the ladder” = 10 to “bottom of the ladder” = 1. Due to high correlations across the five contexts, the mean composite score was computed for analysis (*M* = 1.45, *SD* = 1.21). The Cronbach’s alpha was 0.84.

*Depression*. Depression was measured by the Center for Epidemiologic Studies Depression Scale (Radloff, 1977). Twenty items included: “I was bothered by things that don’t usually bother me.” Responses were coded as “never” = 0, “once in a while” = 1, “some of the time” = 2, “very often” = 3, and “all of the time” = 4. The sum was calculated and used for analysis (*M* = 23.92, *SD* = 12.47). The Cronbach’s alpha was 0.88.

*Anxiety*. Anxiety was measured by the State Trait Anxiety scale (Spielberger et al., 1970). The scale includes 20 items for state anxiety and 20 items for trait anxiety. In this study 20 items for trait anxiety were included, such as “I worry too much over things that don’t really matter” and “I feel nervous and restless.” The responses included: “almost never” = 1, “sometimes” = 2, “often” = 3, and “almost always” = 4. A mean was calculated and used for analysis (*M* = 1.26, *SD* = 0.49). The Cronbach’s alpha was 0.87.

*Discrimination*. Ethnic discrimination was measured by the Racial Ethnic Discrimination Index (REDI; Yip et al., 2019a). Six items were included, such as “I was treated unfairly because of my race/ethnicity over the past 6 months.” The responses were coded as “not at all” = 0, “a little bit” = 1, “somewhat” = 2, “moderately” = 3, “mostly” = 4, “quite a bit” = 5, and “extremely” = 6. A mean score of the six items was used for analysis (*M* = 0.43, *SD* = 0.83). The reliability of this scale from a previous study (Yip et al., 2019a) at the adolescent level was 0.98 and the Cronbach’s alpha from this study was 0.92.

*Self-esteem*. Self-esteem was measured by the Rosenberg Self-Esteem Scale (Rosenberg, 1965). Ten items included: “One the whole, I am satisfied with myself.” The responses were coded as “strongly disagree” = 1, “disagree” = 2, “neutral” = 3, “agree” = 4, and “strongly agree” = 5. A mean was calculated (*M* = 2.50, *SD* = 0.70). The Cronbach’s alpha was 0.86.

*Average grades*. Participants were asked to report the grades from their last report card (i.e., Math, English, Science, and Social Studies/Humanities). Grades were coded as “64 or below” = 1, “69–65” = 2, “74–70” = 3, “79–75” = 4, “84–80” = 5, “89–85” = 6, “94–90” = 7, and “100–95” = 8. An average was calculated (*M* = 4.71, *SD* = 1.58).

*School engagement*. School engagement was measured with an adapted version of the Wellborn measure (Wellborn, 1991). Ten items included: “When I am in class, I participate when we discuss new material.” Responses ranged from “never” = 1 to “all the time” = 5. The mean was 2.35 (*SD* = 0.83), and the Cronbach’s alpha was 0.70.

### Analysis

Step 1: Latent Profile Analysis

Latent profile analysis (LPA) was conducted using *Mplus 7.3* (Muthén and Muthén, 1998-2012). LPA is a person-centered approach useful for detecting subgroups in the sample across multiple variables. This approach is distinguished from the variable-centered approach, which focuses on separate levels on specific variables. A person-centered approach is data-driven analysis which assumes that the subgroups in the sample are not directly observable and infers the associations between observable characteristics. Multiple statistical models are tested and compared to determine the ideal number of latent profiles that represent different patterns across the study variables. LPA provides information about the number of participants that can be classified in each subgroup and an estimate of the level of each variable for each member of the profile.

Latent profile analysis was used to identify the subgroups using adolescents’ ERI exploration and commitment, American identity, and SSS. All variables were standardized to account for differences in measurement scales. The goodness-of-fit was tested with fit indices such as AIC, BIC, ABIC, and entropy. A decrease in AIC, BIC, and ABIC values and entropy values above 0.8 and closer to 1 indicate a better fit. The number of latent profiles is determined by observing the decrease in AIC, BIC, and ABIC values. The number of subgroups at the point where the decrease starts to plateau is selected. The Voung-Lo-Mendell-Rubin Likelihood Ratio Test (VLMR-LRT) and Lo-Mendell-Rubin Adjusted Likelihood Ratio Test (adjusted LMR) examines whether the model with k number of subgroups significantly improves the model fit compared to another model with k−1 number of subgroups (Lo et al., 2001; Tofighi and Enders, 2008). The final number of subgroups are selected based on fit indices and model comparisons with the consideration of interpretability (Lo et al., 2001; Tofighi and Enders, 2008).

Once the social identity profiles have been identified, a chi-square test of independence was conducted to examine the relation between latent profiles and ethnic/racial group (i.e., Asian, Black, and Latinx).

Step 2: Analysis of Variance

Next, differences in adolescents’ discrimination experiences and developmental outcomes across the different LPA profiles were investigated using analysis of variance (ANOVA). In this study, adolescents’ prior experience of discrimination, depression, anxiety, self-esteem, self-reported grades, and school engagement were investigated. While the prior experience of discrimination was measured 14 days before the measurement of all the social identity dimensions, depression, anxiety, self-esteem, self-reported grades, and school engagement were measured after 6 months. Bonferroni *post hoc* test of differences was conducted afterward to identify groups with significantly different means. Although it was difficult to make predictions about their direct causal relationships with the results from ANOVA, the longitudinal design of the current study was expected to provide useful information for future studies that will examine their direct causal relationships.

## Results

### Correlations

Correlation analyses were conducted with the main study variables (Table 1). Adolescents’ age was negatively correlated with American identity, such that older adolescents had higher levels of American identity than younger adolescents. Being male was correlated with higher levels of self-esteem and higher self-reported average grades, lower levels of ERI exploration and anxiety. As for nativity, being born in the United States was associated with higher levels of American identity and lower levels of discrimination experience. Significant correlations were also found between ERI processes and other main study variables, such that ERI exploration was positively correlated with ERI commitment and American identity, and ERI commitment was positively correlated with American identity, self-esteem, and school engagement, while being negatively correlated with discrimination experience, depression, and anxiety. American identity was also found to be positively correlated with self-esteem and negatively correlated with discrimination experience and anxiety. Expectedly, SSS was also positively correlated with self-esteem and school engagement and negatively correlated with anxiety. For mental-health-related outcomes, depression was positively correlated with anxiety and negatively correlated with self-esteem and school engagement. Similarly, anxiety was negatively correlated with self-esteem and school engagement, and self-esteem was positively correlated with school engagement. Lastly, self-reported grades were negatively correlated with school engagement.

**TABLE 1:** Bi-variate correlations of main study variables.

		1	2	3	4	5	6	7	8	9	10	11	12
1	Age	–											
2	Gender	0.04	–										
3	Nativity	0.06	0.13*	–									
4	ERI exploration	–0.02	−0.19**	0.01	–								
5	ERI commitment	–0.08	–0.10	0.01	0.69**	–							
6	American identity	−0.12*	0.08	0.23**	0.14*	0.20**	–						
7	Subjective social status	–0.04	0.05	–0.10	0.03	0.05	0.09	-					
8	Discrimination (pre)	0.08	–0.06	−0.15*	–0.07	−0.11*	−0.11*	–0.06	−				
9	Depression (6m)	–0.02	–0.10	0.02	–0.11	−0.26**	–0.09	–0.08	0.09	–			
10	Anxiety (6m)	0.05	−0.19*	0.04	–0.07	−0.21**	−0.20*	−0.21**	0.21**	0.72**	–		
11	Self-Esteem (6m)	–0.06	0.19*	–0.00	0.09	0.19*	0.20*	0.17*	−0.21**	−0.50**	−0.71**	–	
12	School Engagement (6m)	0.03	0.01	–0.07	0.14	0.18*	–0.00	0.17*	–0.08	−0.30**	−0.33**	0.44**	–
13	Average Grades (6m)	0.09	0.22*	0.13	–0.10	–0.00	0.19	0.09	–0.13	0.17	–0.01	–0.01	−0.30**

* *p* < 0.05, ** *p* < 0.01, 6m = data measured 6-month post.

### Latent Profiles Analysis

**FIGURE 1:**
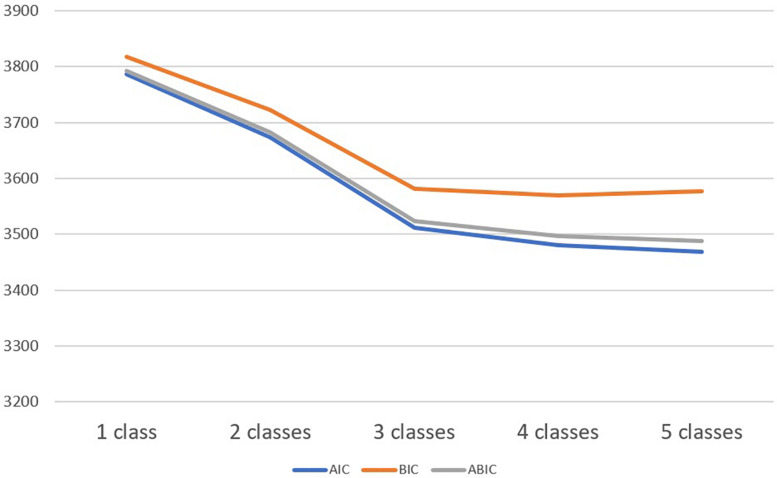
Goodness-of-fit indices.

Based on the fit indices (AIC, BIC, ABIC, and entropy) and tests of model fit (VLMR-LRT and adjusted LMR) and interpretability, a three-profile solution was identified as the ideal LPA solution. According to Table 2, the decrease in the values of AIC, BIC, and ABIC continues and starts to level off for the 3-class model (Figure 1). The entropy for this model was also acceptable. The model comparison between 2-class model and 3-class model indicated that the 3-class model had a significantly better fit than the 2-class model. Based on the fit indices and model fit comparison tests, 3-class model was selected.

**TABLE 2:** Fit indices for latent profile analysis.

Class	AIC	BIC	ABIC	Entropy	VLMRT	ALMRT
1 Class	3786.374	3817.146	3791.768			
2 Classes	3672.816	3722.82	3681.58	0.918	*p* < 0.01	*p* < 0.01
3 Classes	3511.504	3580.74	3523.639	0.891	*p* < 0.001	*p* < 0.001
4 Classes	3481.042	3569.51	3496.547	0.892	n.s.	n.s.
5 Classes	3468.625	3576.325	3487.501	0.868	n.s.	n.s.

**FIGURE 2:**
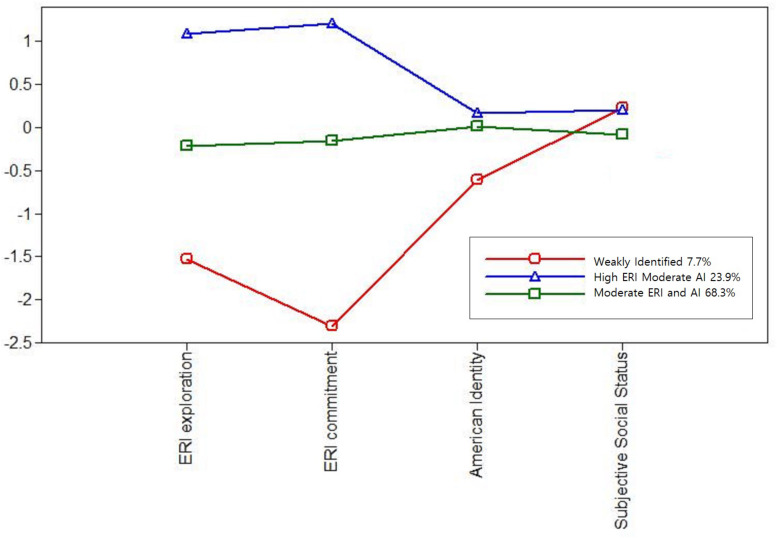
Latent profiles of ERI exploration, ERI commitment, American identity, and subjective social status.

The first profile (Figure 2) consisted of 7.7% of the sample (*n* = 27). This group was characterized by low levels of ERI exploration and commitment, and American identity, and moderate levels of SSS. In this group, 25.9% (*n* = 7) were Asian, 40.7% (*n* = 11) Black, and 33.3% (*n* = 9) Latinx adolescents. Approximately 63.0% (n = 17) were female and 73.9% (*n* = 17) were born in the United States. This group was characterized by, low levels of ERI and American identity and labeled, “weakly identified.”

The second latent profile (Figure 2) consisted of 23.9% (*n* = 77) of the sample. This group was characterized by high levels of ERI, relatively moderate levels of American identity and moderate levels of SSS. The largest ethnic/racial group in this profile was Latinx (42.9%, *n* = 33), followed by Asian (29.9%, *n* = 23) and Black (27.3%, *n* = 21). Similar to the previous profile, most of the participants in this group were female (76.6%, *n* = 59) and born in the United States (77.6%, *n* = 45). This group was labeled, “high ERI moderate AI.”

The third profile (Figure 2) consisted of 68.3% (*n* = 242) of the sample. This group was characterized by moderate levels of ERI, relatively moderate levels of American identity, and moderate levels of SSS. The largest ethnic/racial group in this group was Asian (46.7%, *n* = 113), followed by Latinx (35.5%, *n* = 86) and Black (17.8%, *n* = 43) adolescents. Again, most of the individuals in this group were also female (67.8%, *n* = 164) and born in the United States (76.3%, *n* = 116). This group was labeled, “moderate ERI and AI.”

In order to examine differences in the presence of these profiles across ethnic/racial groups, a chi-square test was conducted. There was a significant relationship between the profiles and adolescents’ ethnicity/race, *X*^2^ (4, *N* = 346) = 13.98, *p* < 0.05 (Table 3). The largest ethnic/racial group in the “weakly identified” group was Black (40.7%), “high ERI moderate AI” was mostly composed of Latinx adolescents (42.9%), while the largest ethnic/racial group in the “moderate ERI and AI” was Asian (46.7%).

**TABLE 3:** Chi-square test for latent classes and race/ethnicity.

		Weakly identified	High ERI Moderate AI	Moderate ERI and AI	Total
Asian	Count	7	23	113	143
	% Within ethnic groups	4.9%	16.1%	79.0%	100.0%
	% Within class	25.9%	29.9%	46.7%	41.3%
Black	Count	11	21	43	75
	% Within ethnic groups	14.7%	28.0%	57.3%	100.0%
	% Within class	40.7%	27.3%	17.8%	21.7%
Latinx	Count	9	33	86	128
	% Within ethnic groups	7.0%	25.8%	67.2%	100.0%
	% Within class	33.3%	42.9%	35.5%	37.0%
Total	Count	27	77	242	346
	% Within ethnic groups	7.8%	22.3%	69.9%	100.0%
	% Within class	100.0%	100.0%	100.0%	100.0%

### Analysis of Variance

Analysis of variance was conducted to examine group differences in social identity dimensions (i.e., ERI, AI, and SSS), as well as discrimination and developmental outcomes of depression, anxiety, school engagement, and grades. The results showed significant differences in ERI exploration, *F*(2, 338) = 180.03, *p* < 0.001, ERI commitment, *F*(2, 337) = 657.91, *p* < 0.001, and AI, *F*(2, 335) = 5.94, *p* < 0.01, across the three profiles (Table 4). The “weakly identified” group showed the lowest levels of ERI exploration (*M*_standardized score_ = −1.52, *SD* = 0.76), ERI commitment (*M*_standardized score_ = −2.30, *SD* = 0.66), and AI (*M*_standardized score_ = −0.57, *SD* = 1.51). The “high ERI moderate AI” group showed higher levels of ERI exploration (*M*_standardized score_ = 1.16, *SD* = 0.64) and commitment (*M*_standardized score_ = 1.25, *SD* = 0.38) than the other two groups and higher levels of AI (*M*_standardized score_ = 0.20, *SD* = 1.17) compared to the “weakly identified” group. The “moderate ERI and AI” group showed higher levels of ERI exploration (*M*_standardized score_ = −0.20, *SD* = 9.71), ERI commitment (*M*_standardized score_ = −0.15, *SD* = 0.45), and AI (*M*_standardized score_ = 0.00, *SD* = 1.00) compared to the “weakly identified” group. However, their ERI exploration and commitment levels were lower than those of the “high ERI moderate AI” group. No significant differences were found for SSS, which will be discussed further in the following section “Discussion.”

**TABLE 4:** Analysis of variance results for three profiles: social identity dimensions.

	ERI exploration *M (SD)*	ERI commitment *M (SD)*	American identity *M (SD)*	SSS *M (SD)*
Weakly identified (*N* = 27)	−1.52 (0.76)*_*a*_*	−2.30 (0.66)*_*a*_*	−0.57 (1.51)*_*a*_*	0.22 (1.40)*_*a*_*
High ERI moderate AI (*N* = 77)	1.16 (0.64)*_*c*_*	1.25 (0.38)*_*c*_*	0.20 (1.17)*_*b*_*	0.19 (1.02)*_*a*_*
Moderate ERI and AI (*N* = 242)	−0.20 (0.71)*_*b*_*	−0.15 (0.45)*_*b*_*	0.00 (1.00)*_*b*_*	−0.08 (0.95)*_*a*_*

*All of the scores have been standardized. Bonferroni *post hoc* test of differences. Different letters (a, b, and c) indicate statistically significant differences across each column.*

The three profiles differed in the levels of prior discrimination experience, *F*(2, 335) = 3.57, *p* < 0.05, and subsequent depressive symptoms, *F*(2, 161) = 3.21, *p* < 0.05, and school engagement, *F*(2, 155) = 3.51, *p* < 0.05 (Table 5). The “weakly identified” group scored the highest on prior discrimination experiences (*M*_weakly identified_ = 0.86, *SD*_weakly identified_ = 1.22; *M*_high ERI moderate AI_ = 0.41, *SD*_high__ERI moderate AI_ = 0.70; *M*_moderate ERI and AI_ = 0.39, *SD*_moderate ERI and AI_ = 0.82) and depression 6 months later (*M*_weakly identified_ = 1.73, *SD*_weakly identified_ = 0.96; *M*_high ERI moderate AI_ = 1.15, *SD*_high ERI moderate AI_ = 0.64; *M*_moderate ERI and AI_ = 1.31, *SD*_moderate ERI and AI_ = 0.69). The “moderate ERI and AI” group displayed the lowest level of school engagement (*M*_moderate ERI and AI_ = 2.60, *SD*_moderate ERI and AI_ = 0.77) compared to the other two groups (*M*_weakly identified_ = 2.65, *SD*_weakly identif__ied_ = 0.73; *M*_high ERI moderate AI_ = 2.89, *SD*_high ERI moderate AI_ = 0.74). The interpretation and implications for these results are discussed in the following section.

**TABLE 5:** Analysis of variance results for three profiles: developmental experiences and outcomes.

Variable	Weakly identified *M (SD)*	High ERI moderate AI *M (SD)*	Moderate ERI and AI *M (SD)*
Prior discrimination	0.86 (1.22)*_*a*_*	0.41 (0.70)*_*b*_*	0.39 (0.82)*_*b*_*
Depression 6 months later	1.73 (0.96)*_*a*_*	1.15 (0.64)*_*b*_*	1.31 (0.69)*_*b*_*
Anxiety 6 months later	1.40 (0.53)*_*a*_*	1.09 (0.52)*_*a*_*	1.23 (0.47)*_*a*_*
Self-esteem 6 months later	2.47 (0.60)*_*a*_*	2.69 (0.61)*_*a*_*	2.52 (0.52)*_*a*_*
School engagement 6 months later	2.65 (0.73)*_*a*_*	2.89 (0.74)*_*a*_*	2.60 (0.77)*_*b*_*
Average grades 6 months later	3.00 (1.99)*_*a*_*	2.26 (1.77)*_*a*_*	2.14 (1.70)*_*a*_*

*Bonferroni *post hoc* test of differences. Different letters (a, b, and c) indicate statistically significant differences across each row.*

## Discussion

As part of social identity development, ethnic/racial minority adolescents in the United States begin to understand who they are in the contexts of their ethnic/racial groups and American society. They also start to develop their subjective sense of social status in relation to these developmental contexts. All of these distinct social identity dimensions – ERI, American identity, and SSS – do not function separately but operate in combination with one another. While previous studies have identified independent roles of these dimensions in adolescent development, there has been a limited understanding in the intersectionality of these dimensions depicted by various configurations exhibiting different developmental implications (Chatman et al., 2001). In this study, ethnic/racial adolescents’ profiles of ERI, American identity, and SSS were explored. Three profiles, “weakly identified,” “high ERI moderate AI,” and “moderate ERI and AI” groups were identified. With the identified profiles, differences in prior experiences of discrimination, and later mental health and academic outcomes were examined. The “weakly identified” group experienced the highest levels of discrimination and depression after 6 months. The “moderate ERI and AI” group reported the lowest levels of school engagement.

The current study’s findings demonstrate the utility of considering various configurations of multiple social identity dimensions in unpacking the development of ethnic/racial minority adolescents. By identifying three distinct identity profile configurations, we observe both between-profile homogeneity and within-profile heterogeneity. Across the three profiles, the average SSS levels were statistically similar. When considered with ERI and American identity, SSS did not display much variability among the adolescents in this study. It is possible that despite the differences in actual social status, the adolescents’ subjective perception was affected by people’s general tendency to view their own group in the positive light in order to maintain their well-being (Alicke, 1985). This tendency may not have been observed in ERI and AI because these dimensions include items about how much they identify with each group and the extent to which each aspect of identification has developed, rather than a mere perception of their standing on a scale. In future studies, it may be helpful to include other measures of perceived social status along with the scale used in the current study in order to gain a multidimensional perspective of adolescents’ perception of their social status. For example, feelings of relative deprivation (Runciman, 1966) as well as a family affluence scale (Currie et al., 2008) could be included. Regardless, including SSS in the analysis contributed to identifying within-group heterogeneity. In other words, we were able to find that despite similarities in SSS levels, different developmental outcomes were observed when it was examined alongside other social identity dimensions.

Although the average levels of SSS were similar across groups, having different configurations of ERI and American identity levels displayed differences in adolescents’ reports of discrimination experience and various developmental outcomes. These results imply that even if adolescents may feel like they belong to similar social statuses, different configurations of ERI and AI are important factors for their adjustment such that considering SSS alone would not be sufficient. Moreover, another example of within-group heterogeneity is that within each profile, adolescents demonstrated similar patterns of ERI, American identity and SSS, but diverse ethnic/racial groups were included in each group, suggesting within-profile heterogeneity. In fact, although the proportions were different, all three ethnic/racial minority groups were found in each profile. From a slightly different angle, this result also implies that *different* levels and configurations of ERI, American identity, and SSS can be observed within the *same* ethnic/racial group, and that *similar* levels and configurations of these social identity dimensions may also be found across *different* ethnic/racial groups. These similarities and differences elucidate the importance of continued efforts to examine the commonly overlooked between-group homogeneity and within-group heterogeneity.

When differences were examined, adolescents identified with three different profiles reported significant differences in prior discrimination experiences, and subsequent mental health and academic outcomes. As hypothesized and consistent with Sue and Sue (2013)’s stages of racial/cultural identity development, adolescents in the “weakly identified” group reported the highest levels of prior discrimination experiences and depression after 6 months (Donovan et al., 2013; Sanchez et al., 2016; Tabbah et al., 2016). They may have been in the dissonance stage where they experienced conflict and shame in identifying with either their ethnic/racial group membership or American group membership. As they started to become aware of their minority status and question dominant society, they may have become more sensitive to instances of discrimination and felt confused about their identity. In turn, this lack of clarity may have contributed to feelings of depression.

This profile needs to be considered with adolescents’ ethnic/racial group membership in mind. The fewest (7.7%) “weakly identified” group was mostly composed of Black adolescents. Despite being small, this group has important implications because they were the most marginalized of the three profiles. The adolescents, especially Black adolescents, who had low levels of both ERI and AI reported the highest levels of discrimination experience and depression. Compared to the other two ethnic/racial groups, the history of Black racial group in the United States is somewhat unique. Many of them may feel that they do not have a particular immigrant ethnic culture to associate their ERI with, although they could still develop ERI based on their racial culture. It should be noted that adolescents who do not hold a strong attachment to their ethnic/racial group membership and have low levels of AI have been found to be especially vulnerable to discrimination and depression.

Next, the “moderate ERI and AI” group demonstrated the lowest level of school engagement, which was unexpected. According to Sue and Sue (2013)’s stages of racial/cultural identity, these adolescents may be in the introspection stage and that may be the reason that their school engagement scores were lower than the “high ERI moderate AI” group, who may be closer to reaching the ideal stage of integrative awareness. In comparison to the “weakly identified” group who were low in both ERI and American identity, “moderate ERI and AI” group may have experienced higher levels of discomfort within the school social context as they started to put more effort and energy into embracing their social contexts as well as both of their social identity dimensions.

Another notable finding is that the “moderate ERI and AI” group (68.3%) was mostly composed of Asian adolescents. For this group, a potential interaction of model minority stereotype (Ford, 1996), which targets the Asians with moderate levels of ERI and AI may have been particularly harmful for their school engagement. Model minority stereotype expects all Asian students to excel academically, and when Asian adolescents’ ERI and AI have only developed moderately at the time that they were going through the introspection stage, they may have been more prone to experiencing pressure from the model minority stereotype and had difficulty engaging at school. However, considering that a good proportion of Latinx adolescents also fell into this profile and that this was the largest profile suggests that there is a need to promote school engagement among a large number of Asian and Latinx adolescents, especially those with moderate levels of ERI and AI.

The “high ERI and moderate AI” group (23.9%) was mostly composed of Latinx adolescents. Adolescents who strongly identified with one’s ethnic/racial group, while moderately associating themselves with the larger American society reported low levels of discrimination experience and depression, and relatively high levels of school engagement. The large Latinx population in the United States and the immigration history that provides a clear sense of where they came from may have contributed to the high ERI and moderate AI. Again, this group also had a good number of both Black and Asian adolescents. This means that for all three ethnic/racial groups, having a high ERI and moderate AI could be beneficial to their development. According to Sue and Sue (2013), they may be on their way to achieving the integrative awareness stage of “high ERI and AI.”

As shown in these findings, while it is important to recognize within-group heterogeneity and between-group homogeneity in subjective social identity dimensions, the objective ethnic/racial segregation and disparities still require continued attention. The quantitative approach of this study provides useful information about the proportion of and average characteristics of adolescents at different intersections of social identity dimensions depicted by various configurations and how each ethnic/racial group is represented within each profile. Particularly, the smallest but most vulnerable “weakly identified” group may not have been identified if the intersectionality of multiple social identity dimensions were not considered quantitatively.

Overall, these findings are consistent with Social Identity Theory (Tajfel and Turner, 1979), which suggests a strong and positive attachment to one’s social groups promotes positive self-image, generating positive mental health, and academic outcomes (Tajfel and Turner, 1986; Cameron, 1999; Smith and Silva, 2011; Rivas-Drake et al., 2014). Adolescents identified with low levels of ethnic/racial and American identity reported higher levels of discrimination experiences and depression after 6 months compared to the other two groups. In other words, adolescents who were weakly identified at the intersection of ERI, American identity, and social status may be more vulnerable than those who had either high levels of ERI and moderate levels of American identity or moderate levels of both ERI and American identity. Even though the “weakly identified” group’s SSS was similar to the SSS levels of the other two groups, this group, with a weak identification with both one’s ethnic/racial group and American group, displayed the highest level of discrimination experiences and subsequent depressive symptoms. Since it is possible that ERI and American identity are reciprocally associated with discrimination (Cheon and Yip, 2019; Zeiders et al., 2019), future studies would benefit from examining their longitudinal causal relationships.

The current study is not without limitations. Future studies would benefit from larger samples to examine longitudinal and direct causal associations. Furthermore, the results are not generalizable because all of the participants lived in and attended schools in New York City, which makes the results and interpretations context specific to the diverse, urban setting. As mentioned earlier, it would also be helpful to include various measures of SSS as the scale used in the current study may not have been sufficient to capture the possible variability.

Despite these limitations, the current study demonstrates one way to operationalize intersectionality as configurations of multiple social identity dimensions. Particularly, the quantitative approach provides information about the approximate proportion and average characteristics of adolescents identified with each profile (Zeiders et al., 2013). Further, the study demonstrates that the inclusion of multiple dimensions of social identity are developmentally relevant and meaningful for adolescents in the United States.

## Data Availability Statement

The datasets generated for this study will not be made publicly available because additional data is still in the process of being collected. Requests to access the datasets should be directed to the corresponding author.

## Ethics Statement

The studies involving human participants were reviewed and approved by Fordham University. Written informed consent to participate in this study was provided by the participants’ legal guardian/next of kin.

## Author Contributions

YC reviewed the literature, conducted statistical analyses, and drafted the manuscript. PI and MH reviewed the literature and drafted the manuscript. TY designed and supervised the original data collection and helped to draft the manuscript. All authors read and approved the final manuscript.

## Conflict of Interest

The authors declare that the research was conducted in the absence of any commercial or financial relationships that could be construed as a potential conflict of interest. The reviewer CB declared a past co-authorship with one of the authors TY.
